# Pleural and mediastinal effusions after the extracardiac total cavopulmonary connection: Risk factors and impact on outcome

**DOI:** 10.3389/fcvm.2022.1026445

**Published:** 2022-11-08

**Authors:** Paul Philipp Heinisch, Paul Metz, Helena Staehler, Benedikt Mayr, Janez Vodiskar, Martina Strbad, Bettina Ruf, Peter Ewert, Alfred Hager, Jürgen Hörer, Masamichi Ono

**Affiliations:** ^1^Department of Congenital and Pediatric Heart Surgery, German Heart Center Munich, Technische Universität München, Munich, Germany; ^2^Division of Congenital and Pediatric Heart Surgery, University Hospital of Munich, Ludwig-Maximilians-Universität, Munich, Germany; ^3^Department of Cardiovascular Surgery, German Heart Center Munich, Technische Universität München, Munich, Germany; ^4^Department of Pediatric Cardiology and Congenital Heart Disease, German Heart Center Munich, Technische Universität München, Munich, Germany

**Keywords:** pleural effusion, univentricular heart, total cavopulmonary connection (TCPC), congenital heart disease, outcome

## Abstract

**Background:**

This study investigated the volume and duration of pleural and mediastinal effusions following extracardiac total cavopulmonary connection, as well as preoperative risk factors and their impact on outcome.

**Materials and methods:**

A total of 210 patients who underwent extracardiac total cavopulmonary connection at our center between 2012 and 2020 were included in this study. Postoperative daily amount of pleural and mediastinal drainage were collected and factors influencing duration and amount of effusions were analyzed. The impact of effusions on adverse events was analyzed.

**Results:**

Median age at extracardiac total cavopulmonary connection was 2.2 (interquartile range, 1.8–2.7) years with median weight of 11.6 (10.7–13.0) kg. Overall duration of drainage after extracardiac total cavopulmonary connection was 9 (6–17) days. The total volume of mediastinal, right pleural, and left pleural drainage was 18.8 (11.9–36.7), 64.4 (27.4–125.9), and 13.6 (0.0–53.5) mL/kg, respectively. Hypoplastic left heart syndrome (*p* = 0.004) and end-diastolic pressure (*p* = 0.044) were associated with high volume of drainages, and hypoplastic left heart syndrome (*p* = 0.007), presence of aortopulmonary collaterals (*p* = 0.002), and high end-diastolic pressure (*p* = 0.023) were associated with long duration of drainages. Dextrocardia was associated with higher volume (*p* < 0.001) and longer duration (*p* = 0.006) of left pleural drainage. Duration of drainage was associated with adverse events following extracardiac total cavopulmonary connection (*p* = 0.015).

**Conclusion:**

Volume and duration of pleural and mediastinal effusions following extracardiac total cavopulmonary connection were related with hypoplastic left heart syndrome, aortopulmonary collaterals, and end-diastolic pressure. The duration of drainage for effusions was a risk factor for adverse events after total cavopulmonary connection.

## Introduction

As patient selection, surgical procedures, and postoperative management have been refined over time, today’s early staged Fontan surgery for patients with univentricular heart is linked to excellent early survival (1–4). Nevertheless, in the absence of a sub-pulmonary ventricle, redirection of the inferior vena cava to the pulmonary circulation inevitably exposes the systemic venous and lymphatic system to significantly higher pressure, which is associated with the majority of the acute and late postoperative complications following the Fontan procedure (5–8). The increase in hydrostatic capillary pressure, which results in excessive filtration in the interstitial space and overwhelming outflow into the lymphatic system, is one of the postulated mechanisms of pleural effusion and chylothorax in Fontan physiology (9).

Our institution has been performing an extracardiac conduit total cavopulmonary connection (EC-TCPC) as the standard procedure (10, 11). Various short-term morbidities may occur following EC-TCPC, including prolonged pleural effusion, arrhythmias, and acute kidney injury, all of which are associated with adverse in-hospital and long-term outcomes (11–14).

In general, studies have consistently found that pleural effusions are associated with morbidity and prolonged hospitalization in EC-TCPC patients (15, 16). Kim et al. were unable to replicate these findings, and prolonged pleural effusions had no effect on late adverse events (17). All possible causes of pleural effusions have been accounted for. A 2019 study conducted at a single center found that prolonged mechanical ventilation can lengthen the time spent in the intensive care unit and the entire hospital stay. A shorter duration of ventilation was also associated with a decreased risk of pleural effusion (18). However, the incidence of postoperative pleural effusions continues to be high and is a significant determinant of postoperative hospital length of stay.

In an effort to close the current knowledge gap, we sought to identify risk factors for pleural effusion and evaluate the effects of pleural and mediastinal effusions following EC-TCPC.

## Materials and methods

### Ethical statement

The Institutional Review Board of the Technical University of Munich approved the study (approved number of 305/20 S-KH on 2nd June, 2020). Due to the retrospective nature of the study, the need for individual patient consent was waived.

### Patients

The retrospective review of medical records including in-hospital and out-patient notes, laboratory data, cardiac catheterization and other non-invasive images was performed for patients who underwent EC-TCPC at the German Heart Center Munich between 2012 and 2020. Patients who underwent TCPC conversion from classic Fontan procedure were excluded. The preoperative variables were measured at the closest time before surgery and postoperative variables were the first measurements when admitted to the ICU. Postoperative daily amounts of right pleural, left pleural and mediastinal effusions from the ICU charts were collected.

### Perioperative management

Following EC-TCPC surgery, all patients were admitted to the intensive care unit (ICU). Extubation was performed several hours after ICU submission. If the drainage of pleural effusion was less than 2 mL/kg/day/tube, the chest/mediastinal tube was removed. The diagnosis of chylothorax was established by fluid analysis through puncture, including the presence of chylomicrons, triglyceride content of ≥110 mg/dl, or a total cell count of 1,000 cells/mm^3^ and lymphocyte fraction ≥80%.

### Operative techniques

Extracardiac conduit total cavopulmonary connection was performed through a median sternotomy on cardiopulmonary bypass with aortic and bicaval cannulation (10, 11). Connection between inferior vena cava and pulmonary artery was performed using a non-ringed Gore-Tex tube graft. A graft diameter of 18 mm was most frequently used. Aortic cross-clamping with subsequent cardioplegic arrest of the heart was done only in patients who received a concomitant intra-cardiac procedure. Fenestration was only created in patients with a high preoperative risk of a poor outcome after TCPC: elevated mean pulmonary artery pressure (>15 mm Hg), elevated pulmonary vascular resistance (>2 mm Hg/L per min/m^2^), pulmonary artery distortion, and elevated ventricular filling pressure (12 mm Hg).

### Follow-up data

The patients underwent outpatient follow-up with pediatric cardiologists, and follow-up periods were defined as the time between the EC-TCPC and the time of the final appointment for each individual patient in the study. Adverse events were defined as death, heart transplantation, TCPC take down, symptomatic protein-losing enteropathy, symptomatic plastic bronchitis, thromboembolic events, sustained episode of supraventricular tachycardia, and requirement of permanent pacemaker implantation after EC-TCPC.

### Statistical analysis

Categorical variables are presented as absolute numbers and percentages. Continuous variables are expressed as medians with interquartile ranges (IQR). Associated factors for excessive volume of drainage after EC-TCPC were identified using a logistic regression analysis. A cut-off value of 75 IQR was used in this analysis. Odds ratio (OR) with 95% confidence interval (CI) were estimated. Cox proportional hazard models was used to identify the variables that are associated with the duration of drainages. Hazard ratio (HR) with 95% CI were estimated. Impact of the duration as well as the amount of effusions on late adverse events after EC-TCPC were analyzed through Cox regression model. Risk factor analysis was tested by a univariable regression model and later as a multivariable model. For the multivariate model, those variables with a *P*-value < 0.1 in the univariable analysis were entered into the multivatiable model, with no subsequent removal of variables. Both forward inclusion and backward elimination process was used to generate the final multivariate model. It was confirmed that the risk factors resulting from forward inclusion and backward elimination was identical. *P*-values < 0.05 were considered significant. Data analysis and graphing were performed with the Statistical Package for the Social Sciences (SPSS) version 28.0 for Windows (IBM, Ehningen, Germany).

## Results

### Patient’s characteristics

A total of 210 Patients were identified who underwent EC-TCPC at our center during the study period. Patient’s characteristics are shown in Table 1. The median follow-up following EC-TCPC was 1.7 (IQR = 0.2–3.8, maximum 8.6) years. Median age at EC-TCPC was 2.2 [Interquartile range (IQR) = 1.8–2.7] years with a body weight of 11.6 (IQR = 10.7–13.0) kg. Most frequent diagnosis was hypoplastic left heart syndrome (*n* = 79, 38%) and dominant right ventricle (RV) was observed in 127 (61%) patients. Dextrocardia was present in 19 patients (9%), and heterotaxy syndrome in 17 (8%). All patients had previous BCPS at the median age of 0.3 (IQR = 0.3–0.5) years and median interval between BCPS and EC-TCPC was 1.8 (IQR = 1.4–2.2) years. Pre-TCPC catheter data is shown in Supplementary Table 1. Operative and perioperative data are depicted in Table 2. An 18 mm extra-cardiac conduit was used in 194 patients (92%).

**TABLE 1:** Patient characteristics.

Variables	ALL (*n* = 210)
	*n* (%) or median (IQR)
Number of patients	210
Age at total cavopulmonary connection (TCPC)	2.2 (1.8–2.7)
Weight at TCPC	11.6 (10.7–13.0)
Male sex	123 (58.6)
**Primary diagnosis**	
Hypoplastic left heart syndrome (HLHS)	79 (37.6)
Univentricular heart (UVH)	30 (14.3)
Tricuspid atresia (TA)	27 (12.9)
Double inlet left ventricle (DILV)	23 (11.0)
Unbalanced atrioventricular septal defect (UAVSD)	12 (5.7)
Congenitally corrected transposition of the great arteries (ccTGA)	10 (4.8)
Pulmonary atresia with intact ventricular septum (PAIVS)	10 (4.8)
Others	19 (9.0)
Dominant right ventricle (RV)	127 (60.5)
**Associated cardiac anomaly**	
Transposition of the great arteries (TGA)	42 (20.0)
Double outlet right ventricle (DORV)	24 (11.4)
Coarctation of the aorta (CoA)	19 (9.0)
Dextrocardia	19 (9.0)
Heterotaxy	17 (8.1)
TAPVC/PAPVC	19 (9.0)
Systemic venous return anomaly	22 (10.5)
**Stage I palliation**	
Norwood/Damus-Kaye-Stansel (DKS) procedure	105 (50.0)
Aortopulmonary shunt (APS)	47 (22.5)
Pulmonary artery banding (PAB)	21 (10.0)
Atrioseptectomy	5 (2.4)
**Stage II palliation**	
Bidirectional cavopulmonary shunt (BCPS)	210 (100.0)
Age at BCPS	0.3 (0.3–0.5)
Interval BCPS and TCPC	1.8 (1.4–2.2)
Other procedures prior to TCPC	
Pacemaker implantation	2 (1.0)
Pulmonary artery reconstruction	48 (22.9)
Atrioventricular valve procedure	32 (15.2)

IQR, interquartile ranges; TAPVC/PAPVC, total (partial) anomalous pulmonary venous connection.

**TABLE 2:** Perioperative variables.

Variables	ALL (*n* = 210)
*n* (%) or median (IQR) or mean ± SD	
Number of patients	210
**Operative data**	
**Conduit diameter (mm)**	
16	3 (1.4)
18	194 (92.4)
20	13 (6.2)
Cardiopulmonary bypass (CPB) time (min)	67 (50–91)
Need for Aortic cross-clamp	39 (18.6)
Aortic cross-clamp time (min)	44 (26–68)
Concomitant procedure	34 (16.2)
Damus-Kaye-Stansel (DKS) procedure	1 (0.5)
Atrioventricular valve (AVV) procedure	20 (9.5)
Pulmonary artery (PA) reconstruction	10 (4.9)
Atrioseptectomy	5 (2.4)
Systemic ventricular outflow tract enlargement	3 (1.4)
Pacemaker implantation	2 (1.0)
Fenestration	4 (1.9)
**Postoperative data**	
Intensive care unit stay (days)	4 (3–7)
Hospital stay (days)	18 (13–29)
Chylothorax	46 (21.9)
Need for ascites drainage	35 (16.7)
Secondary fenestration	2 (1.0)

### Volume and duration of mediastinal and pleural drainage

**FIGURE 1:**
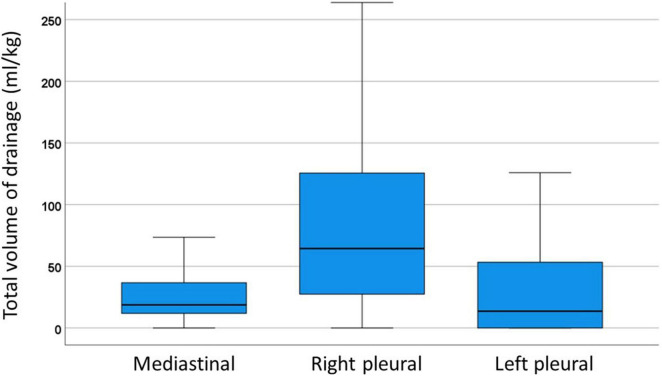
Box-and-whiskers dot plots showing total volume of drainage in right pleural, left pleural and mediastinal drainage. The upper and lower whiskers mark the minimum and maximum values, the upper and lower borders of the box represent the upper and lower quartiles, and the middle horizontal line represents the median.

Median total volume of mediastinal, right pleural, and left pleural drainage after EC-TCPC was 18.8 (IQR, 11.9–36.7), 64.4 (IQR, 27.4–125.9), and 13.6 (0.0–53.5) mL/kg, respectively (Figure 1). Median total volume from any drainage per patient was 120.3 (IQR = 61.0–202.7) mL/kg. Histogram of total volume from any drainage per weight is shown in Supplementary Figure 1.

**FIGURE 2:**
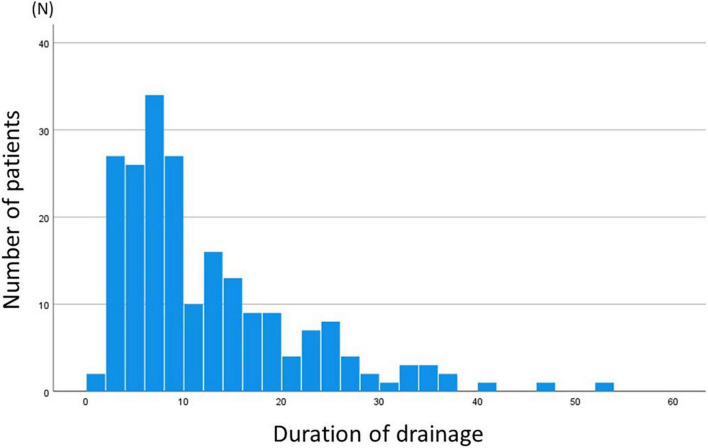
Histogram of duration of drainage. Median duration of drainage was at 9 (IQR = 5–17, minimum 1 and maximum 52) days.

Overall duration of any drainage was 9 (IQR 6–17) days, and the last drainage needed was right pleural drainage in 136 (64.8%), left pleural drainage in 33 (15.7%) patients, and both right and left pleural drainage in 41 (19.5%). Distribution of the duration of the drainage was shown in Figure 2. Median duration of right pleural and left pleural drainage after EC-TCPC was 7 (IQR = 4–13) days, and 6 (IQR = 1–6) days, respectively. Distribution of the duration of right pleural drainage and left pleural drainage is graphically shown in Supplementary Figure 2.

### Factors influencing volume of mediastinal and pleural effusion

As for the total volume of mediastinal and pleural drainage, HLHS (OR = 2.097, *p* = 0.022), presence of APCs (OR = 3.115, *p* = 0.008), PAP (OR = 1.199, *p* = 0.013), SVEDP (OR = 1.199, *p* = 0.011), and systolic arterial pressure (OR = 1.027, *p* = 0.037) were associated with volume from drainage with univariable analysis (Table 3). Multivariable analysis demonstrated HLHS (OR = 3.898, *p* = 0.004) and SVEDP (OR = 1.205, *p* = 0.044) were independent factors associated with high volume of drainages. Winter respiratory viral season of November through March was not identified as a factor influencing volume of mediastinal and pleural effusions (OR = 0.928, *p* = 0.822).

**TABLE 3:** Factors associated with excessive volume of drainage.

Variables	Univariable model			Multivariable model		
	*P*-value	OR	95% CI	*P*-value	OR	95% CI
**Total volume**						
HLHS	0.022	2.097	1.114–3.948	0.004	3.898	1.541–9.859
Dominant RV	0.339	1.374				
APCs	0.008	3.115	1.347–7.201			
PAP	0.013	1.199	1.039–1.385			
EDP	0.011	1.199	1.042–1.380	0.044	1.205	1.005–1.445
AOPs	0.037	1.027	1.002–1.052			
**Right pleural**						
HLHS	<0.001	3.373	1.776–6.408	0.006	3.408	1.428–8.131
Dominant RV	0.043	2.009	1.024–3.943			
APCs	<0.001	4.137	1.785–9.588	0.003	3.799	1.588–9.088
Norwood	0.005	2.522	1.319–4.821			
PAP	0.013	1.199	1.039–1.385			
**Left pleural**						
Dextrocardia	<0.001	6.082	2.253–16.417	<0.001	14.682	3.970–54.294
PAP	0.002	1.253	1.084–1.448	<0.001	1.508	1.240–1.833
EDP	0.006	1.211	1.056–1.389			
AOPs	0.035	1.027	1.002–1.052			
SO2	<0.001	1.131	1.054–1.215	<0.001	1.178	1.080–1.284
**Mediastinal**						
CPB time	0.032	1.010	1.001–1.019			
Bilateral SVC	0.033	3.506	1.105–11.129			
Anomalous SVD	0.007	3.476	1.408–8.580	0.007	4.512	1.524–13.363
Number of previous operation	0.025	1.875	1.084–3.242			
PAP	0.004	1.239	1.071–1.434	0.004	1.289	1.086–1.530
TPG	0.028	1.276	1.026–1.586			

As for right pleural drainage, HLHS (OR = 3.373, *p* < 0.001), presence of APCs (OR = 4.137, *p* < 0.001), and PAP (OR = 1.199, *p* = 0.013), were associated with volume from drainage with univariable analysis. Multivariable analysis identified HLHS (OR = 3.400, *p* = 0.006) and presence of APCs (OR = 3.799, *p* = 0.003) as independent factors associated with high volume of drainages.

As for left pleural drainage, dextrocardia (OR = 6.082, *p* < 0.001), PAP (OR = 1.253, *p* = 0.002), SVEDP (OR = 1.211, *p* = 0.006), systolic aortic pressure (OR = 1.027, *p* = 0.035), and arterial oxygen saturation (OR = 1.131, *p* < 0.001) were associated with a large volume from drainage in the univariable analysis. Multivariable analysis demonstrated dextrocardia (OR = 14.682, *p* < 0.001), PAP (OR = 1.508, *p* < 0.001), and arterial oxygen saturation (OR = 1.178, *p* < 0.001) were independent factors associated with high volume of drainages. Results of the analysis using all variables are shown in Supplementary Table 2. Results of risk factor analysis for total pleural effusions (right and left altogether) are shown in Supplementary Table 3.

### Factors influencing duration of pleural effusions

As for total duration of mediastinal and pleural drainage, HLHS (HR = 1.776, *p* < 0.001), dominant RV (HR = 1.590, *p* = 0.001), presence of APCs (HR = 2.222, *p* < 0.001), previous Norwood procedure (HR = 1.541, *p* = 0.002), PAP (HR = 1.093, *p* = 0.004), and SVEDP (HR = 1.072, *p* = 0.020) were associated with total duration of drainage in univariable analysis (Table 4). Multivariable analysis demonstrated that HLHS (HR = 1.818, *p* = 0.007), presence of APCs (HR = 1.988, *p* = 0.002), and SVEDP (HR = 1.083, *p* = 0.023) were independent factors associated with total duration of drainages. Winter respiratory viral season of November through March was not identified as a factor influencing duration of pleural effusions (HR = 0.980, *p* = 0.890).

**TABLE 4:** Factors associated with duration of drainage.

Variables	Univariable model			Multivariable model		
	*P*-value	HR	95% CI	*P*-value	HR	95% CI
**Total**						
HLHS	<0.001	1,776	1.329–2.340	0.007	1,818	1.177–2.808
Dominant RV	0.001	1,590	1.197–2.109			
APCs	<0.001	2,222	1.485–3.322	0.002	1,988	1.273–3.095
Norwood	0.002	1,541	1.166–2.032			
PAP	0.004	1,093	1.027–1.161			
SVEDP	0.020	1,072	1.011–1.136	0.023	1,083	1.011–1.161
**Right pleural**						
HLHS	<0.001	3,745	1.321–2.358	<0.001	2,088	1.418–3.067
Dominant RV	0.006	1,484	1.119–1.964			
APCs	<0.001	2,128	1.432–3.164	0.003	1,835	1.228–2.739
Norwood	0.002	1,529	1.161–2.012			
PAP	0.010	1,085	1.019–1.153			
**Left pleural**						
Dextrocardia	0.011	1,859	1.152–2.994	0.006	2,105	1.234–3.597
PAP	0.009	1,083	1.020–1.150	0.001	1,111	1.041–1.186
SVEDP	0.016	1,072	1.013–1.133			

As for duration of right pleural drainage, HLHS (HR = 3.745, *p* < 0.001), dominant RV (HR = 1.484, *p* = 0.006), presence of APCs (HR = 2.128, *p* < 0.001), previous Norwood procedure (HR = 1.529, *p* = 0.002), and PAP (HR = 1.085, *p* = 0.010) were associated with long duration of drainage in univariable analysis. Multivariable analysis showed that HLHS (HR = 2.088, *p* < 0.001) and presence of APCs (HR = 1.835, *p* = 0.003) were independent factors associated with long duration of drainages.

As for duration of left pleural drainage, dextrocardia (HR = 1.859, *p* = 0.011), PAP (HR = 1.083, *p* = 0.009), and SVEDP (HR = 1.072, *p* = 0.162), were associated with long duration of drainage in univariable analysis. Multivariable analysis demonstrated that dextrocardia (HR = 2.105, *p* = 0.006) and PAP (HR = 1.111, *p* = 0.001) were independent factors associated with long duration of drainages. The additional surgical procedures did not influence the analyzed outcomes. Results of the analysis using all variables are shown in Supplementary Table 4.

### Impact of amount and duration of mediastinal and pleural drainage on outcome after total cavopulmonary connection

As for the impact of effusion on late outcomes, the amount of effusion was not identified as an increased risk of adverse outcomes following EC-TCPC (Table 5). Whereas total duration of any drainages (HR = 1.074, *p* = 0.022) and duration of left pleural drainage (HR = 1.082, *p* = 0.043) were associated with an increased risk for late adverse outcomes. Need for ascites drainage was also associated with adverse events (HR = 6.768, *p* = 0.036). Variables which influenced the amount and duration of drainage were not associated with the following variables: HLHS (HR = 0.976, *p* = 0.979), presence of APCs (HR = 0.674, *p* = 0.732), previous Norwood procedure (HR = 1.251, *p* = 0.807), PAP (HR = 1.175, *p* = 0.385), SVEDP (HR = 1.248, *p* = 0.292), Dextrocardia (HR = 0.043, *p* = 0.657), systolic arterial pressure (HR = 0.980, *p* = 0.428), arterial oxygen saturation (HR = 0.969, *p* = 0.135), anomalous systemic venous drainage (HR = 0.043, *p* = 0.641), TPG (HR = 1.402, *p* = 0.313), dominant RV (HR = 0.932, *p* = 0.939), and heterotaxy (HR = 0.043, *p* = 0.657). The total duration of any drainage was an independent factor associated with adverse events following EC-TCPC (HR = 1.098, *p* = 0.015) as determined by multivariable analysis. The details of adverse events are shown in Supplementary Table 5.

**TABLE 5:** Factors associated with adverse events after total cavopulmonary connection (TCPC).

Variables	Univariable model			Multivariable model		
	*P*-value	HR	95% CI	*P*-value	HR	95% CI
**Mediastinal and pleural**						
**Volume (mL/kg)**						
Mediastinal (kg)	0.382	1.002	0.998–1.005			
Right pleural (kg)	0.320	1.003	0.997–1.010			
Left pleural (kg)	0.404	1.003	0.996–1.010			
**Duration (days)**						
Total	**0.022**	1.074	1.010–1.141	**0.015**	1.098	1.018–1.184
Mediastinal	0.418	1.066	0.914–1.242			
Right pleural	0.323	1.038	0.964–1.118			
Left pleural	**0.043**	1.082	1.002–1.168			
**Ascites**						
Need for Ascites drainage	**0.036**	6.768	1.129–40.568			

Bold values are statistically significant.

### Chylothorax

Chylothorax was observed in 46 patients (21.9%). Patients with chylothorax had significantly longer duration of right pleural drainage (*p* < 0.001) and left pleural drainage (*p* < 0.001). Volume of drainage was higher in patients with chylothorax in the right pleural drainage (*p* < 0.001) and left pleural drainage (*p* < 0.001).

## Discussion

According to the findings of the current study, HLHS, dominant RV, APCs, PAP, and SVEDP were all associated with longer durations of pleural and mediastinal drainage after EC-TCPC. This was also the case for the volume of drainage that occurred after the procedure. Both the volume and the duration of left pleural drainage were found to be associated with dextrocardia. A risk factor for adverse events that occurred after EC-TCPC was not the amount of effusions themselves, but rather the length of time drainage was performed.

### Factors influencing the amount and duration of pleural and mediastinal drainage

Previous studies demonstrated various factors which influenced postoperative pleural effusions after the Fontan procedure (5–8, 17–22): ventricular stiffness (5), pulmonary atresia (6), pre-Fontan PAP (6, 7, 17, 19, 20), chylothorax (7, 18), atrioventricular valve regurgitation (7), APCs (7, 21, 22), CPB time (7, 8), AXC time (7), oxygen saturation (8), HLHS (17, 23), absence of fenestration (17, 18, 20), younger age at Fontan (18), prolonged mechanical ventilation (18), and winter respiratory viral season of November through March (19). In our study, pre-Fontan PAP and APCs were significant determinants of pleural effusion volume and duration. Risk factors included HLHS, RV dominance, a previous Norwood procedure, APCs, and SVEDP. Dextrocardia was also found to affect the duration and volume of left pleural effusions. In patients with HLHS who had previously undergone the Norwood operation, the volume of right-sided pleural effusion increased significantly. On the contrary, patients with dextrocardia had a significant increase in the duration and volume of left-sided pleural effusion. Though, it is unclear why dextrocardia was linked to the volume and duration of left-sided pleural effusions. In patients with dextrocardia, we assume that the total volume of the left lung is greater than that of the right lung. As a result, the number of left-sided pleural effusions was greater than that of the right lung. However, we do not know why the duration of drainage was longer in the left lung. Further investigation of the pulmonary artery flow profile by cardiac magnetic resonance is required to explain this phenomenon.

The duration of pleural effusion may be affected by the timing of TCPC completion. Our clinical demographic criteria were an age of at least 2 years, a weight of at least ten kilograms, and the ability to walk. In another study, when the chest drainage criteria and age were examined, the factor older age was associated with significantly longer duration of chest drainage (24). According to a recent study, Fontan patients have a reduced carbon monoxide diffusing capacity, which is primarily due to low alveolar volume. Lung stiffness increases, affecting alveolar volume and capillary membrane function. These parameters have a negative correlation with Fontan completion age, implying that earlier Fontan completion may benefit lung function (25). We believe that early Fontan completion might preserve systemic ventricular function, prevent (or at least mitigate) the harmful consequences of the Fontan circulation, and provide better functional ability.

### Impact of the amount and duration of pleural drainage on adverse events

The duration of pleural effusions was found to be related with a higher risk of unfavorable outcomes following the Fontan procedure in several studies (1, 23, 26). Whereas another study did not show the association of adverse outcomes and prolonged pleural effusions (17). According to the findings of our study, the volume of pleural drainage was not a factor in determining whether or not there was an association between EC-TCPC and adverse events.

### Therapeutic options for reducing effusions

Not performing fenestration for EC-TCPC was found to be a risk factor for adverse events including prolonged pleural effusion (6, 17, 18, 20, 27, 28). They demonstrated that fenestration is an effective procedure for lowering pulmonary artery pressure in patients with elevated pulmonary pressure. However, other institutions found no difference in need for chest drains between fenestrated and non-fenestrated Fontan procedure, and adopted selective fenestration strategy (1, 29–31). A propensity score–matched study with 1,443 patients, found no difference in long-term survival or freedom from Fontan failure between patients with and without fenestration (29). Patients with fenestration had a higher incidence of long-term thromboembolic events. It appears that fenestration in Fontan circulation has no long-term benefits. We agree that under usual circumstances a fenestration at the time of an EC-TCPC is not required. Only in patients who were considered to be high-risk, such as those who had an elevated pulmonary artery pressure or trans-pulmonary gradient, significant atrioventricular regurgitation, or single-lung physiology, we choose to perform fenestration (10, 11). In total, we have done EC-TCPC procedures in over 98% of our patients without fenestration.

It is important to recall that a shorter duration of mechanical ventilation is related to reduced pleural effusions. Previous research indicated that early extubation following EC-TCPC, including extubation in the operating room, is a safe and successful approach, resulted in clinical benefits (32–34).

Other therapies include diuretics, surgical and catheter intervention for pathway obstruction, coil embolization of APCs, and secondary fenestration. A defined management strategy might reduce the amount of effusion (35, 36). The use of modified ultrafiltration, which is a standard procedure at our center, helps to reduce postoperative effusions (37).

### Study limitations

This study was limited by its retrospective, non-randomized, and single-center design. Surgical and medical management may have changed during the study period, probably influencing the long-term outcomes. As for adverse events, the pathophysiology leading to the endpoints may be very different, especially for death and rhythm disturbances. Hence, the impact of potential risk factors is difficult to interpret. These might be limitations for this risk analysis. A relatively short follow-up period might cause an unreliability for the analysis of impact of amount and duration of mediastinal and pleural drainage on adverse events.

## Conclusion

According to the findings of our research with a relatively large cohort of non-fenestrated EC-TCPC performed at around 2 years old, pleural effusions following EC-TCPC were linked to a variety of factors, including but not limited to HLHS, RV dominance, Norwood surgery, a high PAP, and a high SVEDP. It was also found that the presence of similar risk factors is associated with greater volumes of pleural effusions. Dextrocardia was an independent factor for prolonged and higher volume of left pleural effusion, suggesting the importance of the dominant lung volume. However, the total amount of effusions was not associated with an increased risk of adverse outcomes, whereas the duration of pleural drainage affects the long-term prognosis following EC-TCPC. In the days following EC-TCPC, patients with persistent pleural effusions must be thoroughly observed.

## Data availability statement

The raw data supporting the conclusions of this article will be made available by the authors, without undue reservation.

## Ethics statement

The studies involving human participants were reviewed and approved by the Institutional Review Board of the Technical University of Munich approved the study (approved number of 305/20 S-KH on 2nd June, 2020). Written informed consent to participate in this study was provided by the participants’ legal guardian/next of kin.

## Author contributions

PH, PM, HS, BM, AH, JH, and MO: conceptualization. PH, PM, AH, JH, and MO: methodology. PH, PM, HS, AH, JH, and MO: validation and writing—review and editing. PH, PM, HS, BM, JV, MS, BR, PE, AH, JH, and MO: resources and writing—original draft. PH, PM, HS, JH, and MO: visualization. JH and MO: supervision and project administration. All authors have read and agreed to the published version of the manuscript.

## Supplementary material

Supplementary Figure 1 Histogram of volume of drainage. Total volume from any drainage per patient was median 120.3 (IQR 61.0–202.7) mL/kg.

Supplementary Figure 2 Histogram of duration of drainage. Median duration of right pleural and left pleural drainage after EC-TCPC was 7 (IQR 4–13, minimum 0 and maximum 51) days, and 2 (IQR 0–7, minimum 0 and maximum 40) days, respectively.
