# Supplementary figures and images for: Pleural and mediastinal effusions after the extracardiac total cavopulmonary connection: Risk factors and impact on outcome
Source: Front Cardiovasc Med. 2022 Nov 8;9:1026445. doi: 10.3389/fcvm.2022.1026445 (PMC9678908; doi:10.3389/fcvm.2022.1026445)

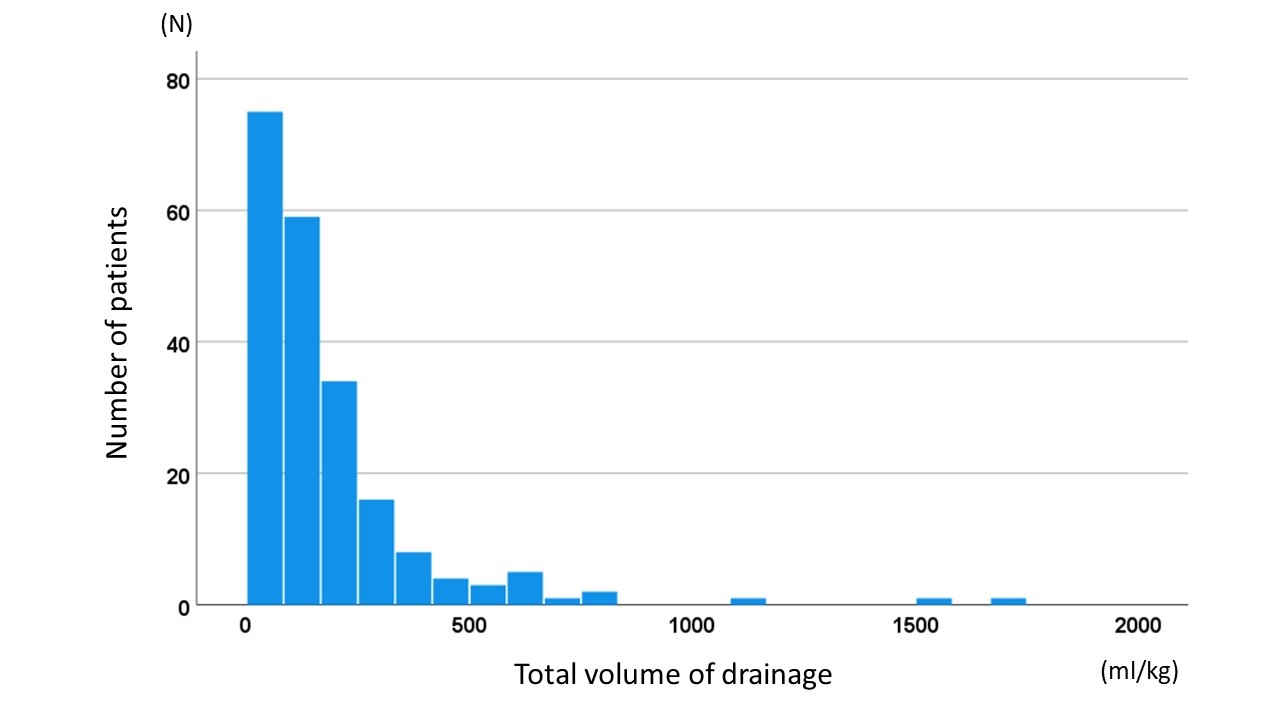

Supplement: Supplementary Figure 1 — Histogram of volume of drainage. Total volume from any drainage per patient was median 120.3 (IQR 61.0–202.7) mL/kg. [file Image_1.JPEG]

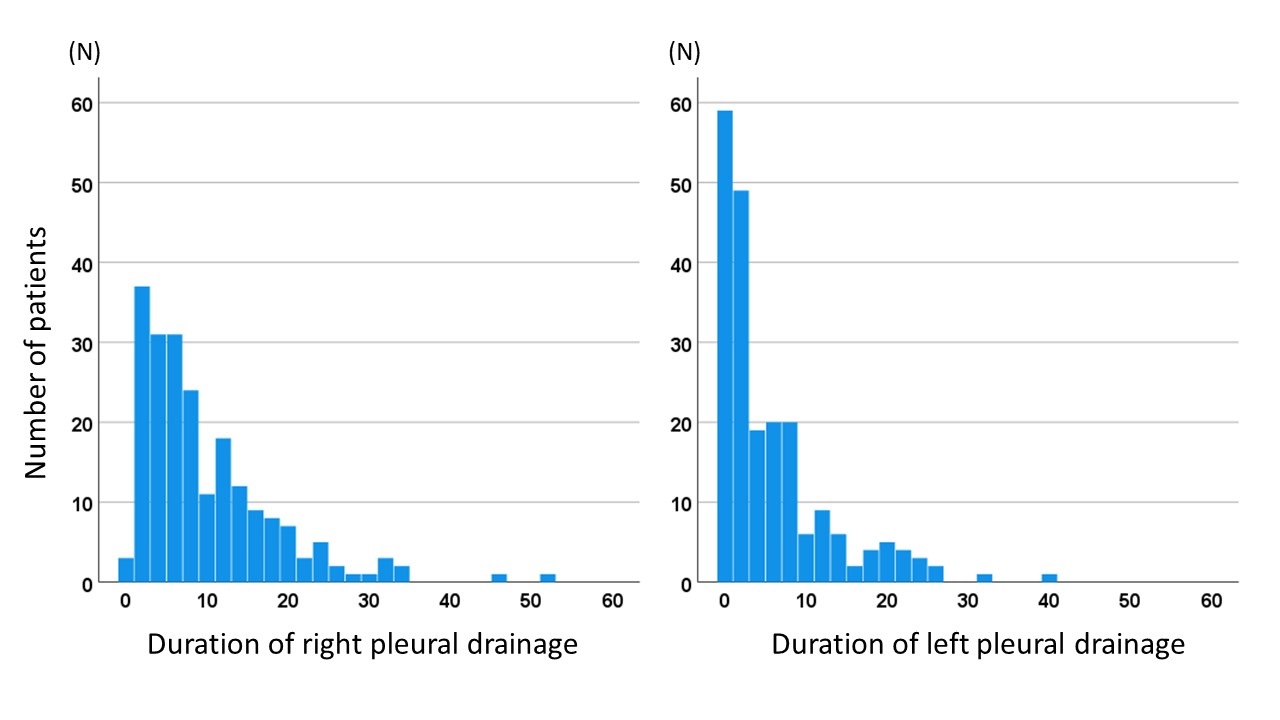

Supplement: Supplementary Figure 2 — Histogram of duration of drainage. Median duration of right pleural and left pleural drainage after EC-TCPC was 7 (IQR 4–13, minimum 0 and maximum 51) days, and 2 (IQR 0–7, minimum 0 and maximum 40) days, respectively. [file Image_2.JPEG]
