# Supplementary material for: Pleural and mediastinal effusions after the extracardiac total cavopulmonary connection: Risk factors and impact on outcome
Source: Front Cardiovasc Med. 2022 Nov 8;9:1026445. doi: 10.3389/fcvm.2022.1026445 (PMC9678908; doi:10.3389/fcvm.2022.1026445)
Supplement: Supplementary file 3 [file Data_Sheet_1.docx]

## Supplementary Table 1

| Supplementary Table 1: Pre-TCPC catheter data | | |
| --- | --- | --- |
| Variables | |  |
|  |  |  |
| Pre-TCPC catheter data | |  |
|  | Hemoglobin (g/dl) | 15.6 (14.7-16.8) |
|  | Mean pulmonary artery pressure (mmHg) | 10 (8-12) |
|  | Mean left atrium pressure (mmHg) | 6 (5-8) |
|  | Transpulmonary gradient (mmHg) | 4 (3-5) |
|  | Systolic ventricular pressure (mmHg) | 87 (81-97) |
|  | Ventricular end-diastolic pressure (mmHg) | 8 (7-10) |
|  | Systolic aortic pressure (mmHg) | 87 (78-96) |
|  | Mean aortic pressure (mmHg) | 61 (55-67) |
|  | Aortic oxygen saturation (%) | 83 (79-86) |
|  | |  |
|  |  |  |
|  |  |  |
|  | |  |

## Supplementary Table 2

| Supplementary Table 2: Factors associated excessive volume of drainage | | | | | | | | | | |  |
| --- | --- | --- | --- | --- | --- | --- | --- | --- | --- | --- | --- |
| Variables | Total | |  | Right pleural | |  | Left pleural | |  | Mediastinal | |
|  | P-value | OR |  | P-value | OR |  | P-value | OR |  | P-value | OR |
| Age at TCPC | 0.207 | 0.837 |  | 0.192 | 0.829 |  | 0.354 | 0.921 |  | 0.809 | 1.011 |
| Weight at TCPC | 0.153 | 0.906 |  | 0.153 | 0.907 |  | 0.420 | 0.970 |  | 0.781 | 0.992 |
| HLHS | 0.022 | 2.097 |  | <0,001 | 3.373 |  | 0.382 | 1.325 |  | 0.316 | 1.383 |
| ccTGA | 0.280 | 0.316 |  | 0.751 | 1.252 |  | 0.270 | 0.308 |  | 0.697 | 0.730 |
| UVH | 0.114 | 0.411 |  | 0.044 | 0.281 |  | 0.897 | 1.059 |  | 0.477 | 0.707 |
| TA | 0.190 | 0.476 |  | 0.174 | 0.463 |  | 0.363 | 0.622 |  | 0.392 | 0.639 |
| DILV | 0.268 | 1.683 |  | 0.965 | 1.022 |  | 0.295 | 1.635 |  | 0.921 | 1.051 |
| PAIVS | 0.723 | 1.286 |  | 0.270 | 0.308 |  | 0.673 | 0.712 |  | 0.280 | 2.054 |
| UAVSD | 0.486 | 0.576 |  | 0.466 | 0.562 |  | 0.466 | 0.562 |  | 0.486 | 0.576 |
| TGA | 0.304 | 0.643 |  | 0.478 | 0.745 |  | 0.752 | 0.881 |  | 0.812 | 0.908 |
| DORV | 0.139 | 0.389 |  | 0.128 | 0.378 |  | 0.562 | 0.736 |  | 0.310 | 0.559 |
| CoA | 0.083 | 2.360 |  | 0.250 | 1.787 |  | 0.093 | 2.292 |  | 0.506 | 1.414 |
| Dextrocardia | 0.228 | 1.839 |  | 0.541 | 1.375 |  | <0.001 | 6.082 |  | 0.910 | 1.064 |
| Heterotaxy | 0.866 | 0.904 |  | 0.432 | 0.597 |  | 0.350 | 1.648 |  | 0.866 | 0.904 |
| Dominant RV | 0.339 | 1.374 |  | 0.043 | 2.009 |  | 0.163 | 1.596 |  | 0.732 | 0.895 |
| CPB time | 0.261 | 1.005 |  | 0.345 | 1.004 |  | 0.093 | 1.008 |  | 0.032 | 1.010 |
| Need AXC | 0.949 | 1.026 |  | 0.412 | 0.701 |  | 0.230 | 1.585 |  | 0.093 | 1.895 |
| Concomitant | 0.497 | 0.733 |  | 0.912 | 1.048 |  | 0.457 | 0.712 |  | 0.541 | 1.289 |
| Bil. SVC | 0.245 | 2.010 |  | 0.914 | 0.929 |  | 0.269 | 1.936 |  | 0.033 | 3.506 |
| APVD | 0.228 | 1.839 |  | 0.541 | 1.375 |  | 0.093 | 2.292 |  | 0.506 | 1.414 |
| ASVD | 0.816 | 1.125 |  | 0.735 | 0.834 |  | 0.232 | 1.764 |  | 0.007 | 3.476 |
| APCs | 0.008 | 3.115 |  | <0.001 | 4.137 |  | 0.075 | 2.095 |  | 0.299 | 1.697 |
| No. of palliation | 0.375 | 1.272 |  | 0.245 | 1.368 |  | 0.393 | 0.781 |  | 0.025 | 1.875 |
| PAB | 0.371 | 1.554 |  | 0.752 | 1.175 |  | 0.752 | 1.175 |  | 0.711 | 1.209 |
| APS | 0.138 | 0.546 |  | 0.138 | 0.546 |  | 0.322 | 0.692 |  | 0.160 | 0.547 |
| Norwood | 0.082 | 1.753 |  | 0.005 | 2.522 |  | 0.344 | 1.350 |  | 0.634 | 1.164 |
| Prior ASE | 0.874 | 0.939 |  | 0.937 | 0.969 |  | 0.937 | 0.969 |  | 0.342 | 0.697 |
| Prior PA repair | 0.285 | 1.497 |  | 0.614 | 0.823 |  | 0.614 | 0.823 |  | 0.674 | 0.849 |
| Prior AVV repair | 0.362 | 0.643 |  | 0.920 | 0.957 |  | 0.590 | 0.780 |  | 0.181 | 0.502 |
| PAP | 0.013 | 1.199 |  | 0.013 | 1.199 |  | 0.002 | 1.253 |  | 0.004 | 1.239 |
| LAP | 0.217 | 1.098 |  | 0.196 | 1.104 |  | 0.092 | 1.135 |  | 0.171 | 1.111 |
| TPG | 0.193 | 1.148 |  | 0.155 | 1.162 |  | 0.225 | 1.136 |  | 0.028 | 1.276 |
| EDP | 0.011 | 1.199 |  | 0.135 | 1.111 |  | 0.006 | 1.211 |  | 0.204 | 1.092 |
| SVP | 0.067 | 1.024 |  | 0.148 | 1.019 |  | 0.082 | 1.023 |  | 0.326 | 1.013 |
| AOPs | 0.037 | 1.027 |  | 0.076 | 1.022 |  | 0.035 | 1.027 |  | 0.210 | 1.015 |
| AoSO2 | 0.122 | 1.051 |  | 0.627 | 1.012 |  | <0.001 | 1.131 |  | 0.700 | 0.993 |

OR; odds ratio, TCPC; total cavopulmonary connection, HLHS; hypoplastic left heart syndrome, ccTGA; congenitally corrected transposition of the great arteries, UVH; univentricular heart, TA; tricuspid atresia, DILV; double inlet left ventricle, PAIVS; pulmonary atresia with intact ventricular septum, UAVSD; unbalanced atrioventricular septal defect, TGA; transposition of the great arteries, DORV; double outlet right ventricle, CoA; coarctation of the aorta, RV; right ventricle, CPB; cardiopulmonary bypass time, AXC; aortic cross clamp, Bil; bilateral, APVD; anomalous pulmonary venous drainage, ASVD; anomalous systemic venous drainage, APCs; aortopulmonary collateral arteries, PAB; pulmonary artery banding, APS; aortopulmonary shunt, ASE; atrioseptectomy, PA; pulmonary artery AVV; atrioventricular valve, PAP; pulmonary artery pressure, LAP; left atrial pressure, TPG; transpulmonary gradient, EDP; end diastolic pressure, SVP; systemic ventricular pressure, AOPs; systolic aortic pressure, AoSO2; aortic oxygen saturation

## Supplementary Table 3

| Supplementary Table 3: Factors associated with total pleural effusion (right + left pleural effusions) | | | | | | | |
| --- | --- | --- | --- | --- | --- | --- | --- |
| Variables |  | | | | | | |
|  | Univariable model | | |  | Multivariable model | | |
|  | P-value | OR | 95% CI |  | P-value | OR | 95% CI |
| Age at TCPC | 0.647 | 0.973 |  |  |  |  |  |
| Weight at TCPC | 0.594 | 0.983 |  |  |  |  |  |
| HLHS | 0.022 | 2.723 | 1.434-5.174 |  | 0.033 | 2.868 | 1.091-7.540 |
| ccTGA | 0.291 | 0.325 |  |  |  |  |  |
| UVH | 0.515 | 0.728 |  |  |  |  |  |
| TA | 0.207 | 0.489 |  |  |  |  |  |
| DILV | 0.506 | 1.381 |  |  |  |  |  |
| PAIVS | 0.291 | 0.325 |  |  |  |  |  |
| UAVSD | 0.205 | 0.262 |  |  |  |  |  |
| TGA | 0.340 | 0.663 |  |  |  |  |  |
| DORV | 0.334 | 0.575 |  |  |  |  |  |
| CoA | 0.472 | 1.455 |  |  |  |  |  |
| Dextrocardia | 0.472 | 1.455 |  |  |  |  |  |
| Heterotaxy | 0.902 | 0.929 |  |  |  |  |  |
| Dominant RV | 0.034 | 2.104 | 1.057-4.190 |  |  |  |  |
| CPB time | 0.337 | 1.004 |  |  |  |  |  |
| Need AXC | 0.787 | 0.893 |  |  |  |  |  |
| concomitant | 0.856 | 0.923 |  |  |  |  |  |
| Bil. SVC | 0.610 | 1.377 |  |  |  |  |  |
| APVD | 0.073 | 2.430 | 0.920-6.416 |  |  |  |  |
| ASVD | 0.773 | 1.158 |  |  |  |  |  |
| APCs | 0.028 | 2.489 | 1.103-5.615 |  |  |  |  |
| No. of palliation | 0.573 | 1.165 |  |  |  |  |  |
| PAB | 0.670 | 1.243 |  |  |  |  |  |
| APS | 0.366 | 0.715 |  |  |  |  |  |
| Norwood | 0.057 | 1.863 | 0.982-3.534 |  |  |  |  |
| Prior ASE | 0.873 | 1.067 |  |  |  |  |  |
| Prior PA repair | 0.275 | 0.639 |  |  |  |  |  |
| Prior AVV repair | 0.973 | 1.015 |  |  |  |  |  |
| PAP | <0.001 | 1.288 | 1.108-1.497 |  | 0.022 | 1.275 | 1.036-1.569 |
| LAP | 0.041 | 1.170 | 1.006-1.360 |  |  |  |  |
| TPG | 0.218 | 1.138 |  |  |  |  |  |
| EDP | 0.004 | 1.232 | 1.067-1.421 |  |  |  |  |
| SVP | 0.128 | 1.020 |  |  |  |  |  |
| AOPs | 0.043 | 1.026 | 1.001-1.052 |  |  |  |  |
| AoSO2 | 0.077 | 1.060 | 0.994-1.130 |  |  |  |  |

OR; odds ratio, TCPC; total cavopulmonary connection, HLHS; hypoplastic left heart syndrome, ccTGA; congenitally corrected transposition of the great arteries, UVH; univentricular heart, TA; tricuspid atresia, DILV; double inlet left ventricle, PAIVS; pulmonary atresia with intact ventricular septum, UAVSD; unbalanced atrioventricular septal defect, TGA; transposition of the great arteries, DORV; double outlet right ventricle, CoA; coarctation of the aorta, RV; right ventricle, CPB; cardiopulmonary bypass time, AXC; aortic cross clamp, Bil; bilateral, APVD; anomalous pulmonary venous drainage, ASVD; anomalous systemic venous drainage, APCs; aortopulmonary collateral arteries, PAB; pulmonary artery banding, APS; aortopulmonary shunt, ASE; atrioseptectomy, PA; pulmonary artery AVV; atrioventricular valve, PAP; pulmonary artery pressure, LAP; left atrial pressure, TPG; transpulmonary gradient, EDP; end diastolic pressure, SVP; systemic ventricular pressure, AOPs; systolic aortic pressure, AoSO2; aortic oxygen saturation

Supplementary Table 4

| Supplementary Table 4: Factors associated with duration of drainage | | | | | | | | | |  |  |
| --- | --- | --- | --- | --- | --- | --- | --- | --- | --- | --- | --- |
| Variables | Total | |  | Right pleural | |  | Left pleural | |  | Mediastinal | |
|  | P-value | HR |  | P-value | HR |  | P-value | HR |  | P-value | HR |
| Age TCPC | 0.820 | 0,995 |  | 0.367 | 0,980 |  | 0.895 | 1,003 |  | 0.614 | 1,010 |
| Weight at TCPS | 0.736 | 0,996 |  | 0.376 | 0,989 |  | 0.872 | 1,002 |  | 0.664 | 1,005 |
| HLHS | <0.001 | 1,776 |  | <0.001 | 3,745 |  | 0.838 | 1,030 |  | 0.211 | 1,198 |
| ccTGA | 0.234 | 0,679 |  | 0.329 | 0,727 |  | 0.032 | 0,494 |  | 0.595 | 0,842 |
| UVH | 0.398 | 0,845 |  | 0.093 | 0,716 |  | 0.377 | 1,192 |  | 0.244 | 0,793 |
| TA | 0.105 | 0,714 |  | 0.191 | 0,763 |  | 0.879 | 1,032 |  | 0.656 | 0,912 |
| DILV | 0.827 | 0,952 |  | 0.929 | 0,980 |  | 0.495 | 1,163 |  | 0.960 | 1,011 |
| PAIVS | 0.003 | 0,382 |  | <0.001 | 0,282 |  | 0.171 | 0,640 |  | 0.512 | 1,238 |
| UAVSD | 0.027 | 0,513 |  | 0.077 | 0,589 |  | 0.293 | 0,729 |  | 0.443 | 0,795 |
| TGA | 0.100 | 0,750 |  | 0.173 | 0,789 |  | 0.426 | 0,871 |  | 0.328 | 0,844 |
| DORV | 0.354 | 0,818 |  | 0.111 | 0,705 |  | 0.593 | 0,890 |  | 0.120 | 0,711 |
| CoA | 0.761 | 0,929 |  | 0.263 | 0,761 |  | 0.953 | 0,985 |  | 0.336 | 1,263 |
| Dextrocardia | 0.614 | 1,130 |  | 0.835 | 1,052 |  | 0.011 | 1,859 |  | 0.462 | 0,838 |
| Heterotaxy | 0.352 | 0,789 |  | 0.148 | 0,692 |  | 0.549 | 1,166 |  | 0.351 | 0,789 |
| Dominant RV | 0.001 | 1,590 |  | 0.006 | 1,484 |  | 0.577 | 1,082 |  | 0.771 | 0,960 |
| CPB time | 0.689 | 1,001 |  | 0.612 | 1,001 |  | 0.306 | 1,002 |  | 0.478 | 1,002 |
| Need AXC | 0.396 | 0,858 |  | 0.139 | 0,767 |  | 0.669 | 1,079 |  | 0.795 | 1,047 |
| Concomitant | 0.791 | 0,951 |  | 0.972 | 0,993 |  | 0.413 | 0,858 |  | 0.724 | 0,935 |
| Bil. SVC | 0.094 | 1,667 |  | 0.334 | 1,339 |  | 0.257 | 1,389 |  | 0.958 | 1,015 |
| APVD | 0.341 | 1,258 |  | 0.366 | 1,244 |  | 0.169 | 1,401 |  | 0.534 | 0,860 |
| ASVD | 0.747 | 1,075 |  | 0.727 | 1,082 |  | 0.149 | 1,391 |  | 0.742 | 1,078 |
| APCs | <0.001 | 2,222 |  | <0.001 | 2,128 |  | 0.228 | 1,259 |  | 0.798 | 1,049 |
| No. of palliation | 0.815 | 1,031 |  | 0.886 | 0,981 |  | 0.506 | 0,923 |  | 0.123 | 1,217 |
| PAB | 0.901 | 1,029 |  | 0.946 | 0,984 |  | 0.154 | 1,403 |  | 0.886 | 0,967 |
| APS | 0.001 | 0,613 |  | 0.001 | 0,618 |  | 0.868 | 1,026 |  | 0.357 | 0,872 |
| Norwood | 0.002 | 1,541 |  | 0.002 | 1,529 |  | 0.653 | 0,939 |  | 0.719 | 1,052 |
| Prior ASE | 0.801 | 1,045 |  | 0.267 | 1,212 |  | 0.756 | 0,948 |  | 0.733 | 0,943 |
| Prior PA repair | 0.531 | 0,901 |  | 0.812 | 1,041 |  | 0.383 | 0,866 |  | 0.212 | 0,812 |
| Prior AVV repair | 0.858 | 1,035 |  | 0.467 | 1,152 |  | 0.942 | 1,014 |  | 0.283 | 0,812 |
| PAP | 0.004 | 1,093 |  | 0.010 | 1,085 |  | 0.009 | 1,083 |  | 0.434 | 1,026 |
| LAP | 0.139 | 1,052 |  | 0.203 | 1,044 |  | 0.326 | 1,035 |  | 0.312 | 1,036 |
| TPG | 0.391 | 1,042 |  | 0,588 | 1,026 |  | 0.268 | 1,055 |  | 0.443 | 1,036 |
| EDP | 0.020 | 1,072 |  | 0.103 | 1,049 |  | 0.016 | 1,072 |  | 0.151 | 1,045 |
| SVP | 0.905 | 0,999 |  | 0.923 | 1,000 |  | 0.410 | 1,005 |  | 0.371 | 1,005 |
| AOPs | 0.601 | 1,013 |  | 0.412 | 1,004 |  | 0.144 | 1,008 |  | 0.191 | 1,007 |
| AoSO2 | 0.220 | 0,987 |  | 0.459 | 0,992 |  | 0.109 | 1,012 |  | 0.515 | 1,006 |

HR; hazard ratio, TCPC; total cavopulmonary connection, HLHS; hypoplastic left heart syndrome, ccTGA; congenitally corrected transposition of the great arteries, UVH; univentricular heart, TA; tricuspid atresia, DILV; double inlet left ventricle, PAIVS; pulmonary atresia with intact ventricular septum, UAVSD; unbalanced atrioventricular septal defect, TGA; transposition of the great arteries, DORV; double outlet right ventricle, CoA; coarctation of the aorta, RV; right ventricle, CPB; cardiopulmonary bypass time, AXC; aortic cross clamp, Bil; bilateral, APVD; anomalous pulmonary venous drainage, ASVD; anomalous systemic venous drainage, APCs; aortopulmonary collateral arteries, PAB; pulmonary artery banding, APS; aortopulmonary shunt, ASE; atrioseptectomy, PA; pulmonary artery AVV; atrioventricular valve, PAP; pulmonary artery pressure, LAP; left atrial pressure, TPG; transpulmonary gradient, EDP; end diastolic pressure, SVP; systemic ventricular pressure, AOPs; systolic aortic pressure, AoSO2; aortic oxygen saturation

Supplementary Table 5

| Supplementary Table 5. Adverse events following EC-TCPC | | | |  |  |
| --- | --- | --- | --- | --- | --- |
| Case | Diagnosis | Age at TCPC (year) | Adverse event | Onset after TCPC (years) | Prognosis |
| 1 | PA, hypo RV | 9 | Thrombus in SVC | 10 | alive |
| 2 | HLHS | 2 | PLE | 2 | alive |
| 3 | TA Ib | 2 | Virus infection | postoperative | death (3 months) |
| 4 | MA, DORV | 2 | MOF | postoperative | death (2 months) |
| 5 | HLHS | 2 | Pacemaker implantation | postoperative | alive |

EC-TCPC, extracardiac total cavopulmonary conneston; PA, pulmonary atresita; RV, right ventricle; HLHS, hypoplastic left heart syndrome; TA, tricuspid atresia; MA, mitral atresia; DORV, double outlet right ventricle; SVC, superior vena cava; PLE, protein losing enteropathy; MOF, multiple organ failure.
